# Statin Treatment Increases Lifespan and Improves Cardiac Health in *Drosophila* by Decreasing Specific Protein Prenylation

**DOI:** 10.1371/journal.pone.0039581

**Published:** 2012-06-21

**Authors:** Stephen R. Spindler, Rui Li, Joseph M. Dhahbi, Amy Yamakawa, Patricia Mote, Rolf Bodmer, Karen Ocorr, Renee T. Williams, Yinsheng Wang, Kenneth P. Ablao

**Affiliations:** 1 Department of Biochemistry, University of California Riverside, Riverside, California, United States of America; 2 Development and Aging Program, Sanford-Burnham Medical Research Institute, La Jolla, California, United States of America; 3 Department of Chemistry, University of California Riverside, Riverside, California, United States of America; Virginia Commonwealth University Medical Center, United States of America

## Abstract

Statins such as simvastatin are 3-hydroxy-3-methylglutaryl coenzyme A (HMG-CoA) reductase inhibitors and standard therapy for the prevention and treatment of cardiovascular diseases in mammals. Here we show that simvastatin significantly increased the mean and maximum lifespan of *Drosophila melanogaster* (*Drosophila*) and enhanced cardiac function in aging flies by significantly reducing heart arrhythmias and increasing the contraction proportion of the contraction/relaxation cycle. These results appeared independent of internal changes in ubiquinone or juvenile hormone levels. Rather, they appeared to involve decreased protein prenylation. Simvastatin decreased the membrane association (prenylation) of specific small Ras GTPases in mice. Both farnesyl (L744832) and type 1 geranylgeranyl transferase (GGTI-298) inhibitors increased *Drosophila* lifespan. These data are the most direct evidence to date that decreased protein prenylation can increase cardiac health and lifespan in any metazoan species, and may explain the *pleiotropic* (non-cholesterol related) health effects of statins.

## Introduction

**Figure 1:**
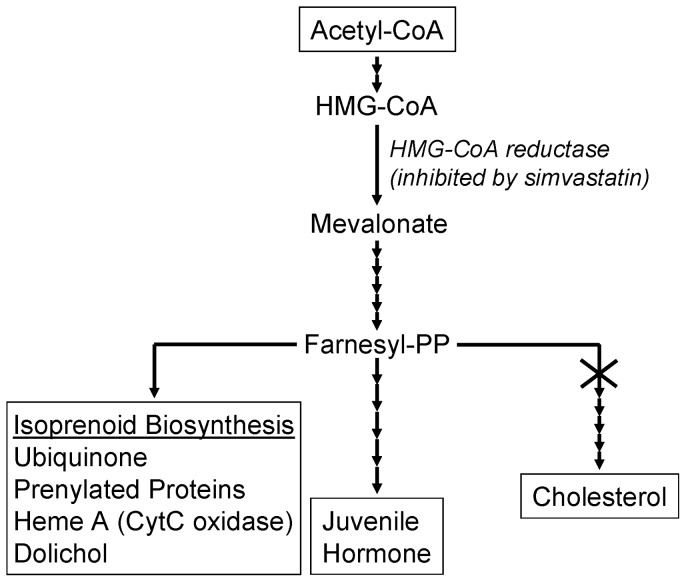
The acetyl CoA-mevalonate pathway in insects and mammals. The X at the head of the cholesterol branch of the pathway indicates that insects lack several enzymes required for cholesterol biosynthesis.

Cardiovascular disease is the leading cause of morbidity and mortality worldwide. Statins, which are competitive inhibitors of HMG-CoA reductase, reduce the biosynthesis of mevalonate. Mevalonate is a precursor in the synthesis of many different compounds in mammals, including cholesterol, isoprenoids, and ubiquinones (Figure 1) [1]. Statins are standard therapies for the prevention and treatment of hyperlipidemia, hypertension, congestive heart failure, and renal disease [2]. A major part of their positive effects on cardiovascular disease arises from a reduction in cholesterol biosynthesis, and an accompanying decrease in plasma cholesterol levels.

Statins also appear to reduce all-cause mortality in healthy, normolipidemic adults [3]. Isoprenoids, which are synthesized downstream of mevalonate, are required for the posttranslational farnesylation and geranylgeranylation of proteins, especially the small, signaling Ras GTPases [1], [4], [5]. Isoprenoid adducts on these signaling GTPases anchor them to cell membranes, which is essential to their activity [6], [7]. Statins reduce the prenylation of Ras and Rho in cell culture, leading to the accumulation of their inactive forms in the cytoplasm [8]. Based mostly on such in vitro studies, reduced prenylation of these small, monomeric GTP binding proteins is postulated to be responsible for many of the non-cholesterol-related health effects of statins [8], [9].

*Drosophila* can be used to study the etiology and treatment of human diseases (e.g. [10], [11]). For example, many of the genetic and metabolic pathways involved in the development and functioning of the heart are evolutionarily conserved between flies and humans [11], [12], as are many of the signaling pathways associated with longevity [13], [14]. Insects lack several of the enzymes required for the *de novo* biosynthesis of cholesterol [15]. Here, we used *Drosophila* as a model for studies of the noncholesterol-related longevity and health effects of statins. Together our data show, more directly than has been done previously, that statins extend lifespan and enhance cardiac function by reducing protein prenylation.

## Results

### Simvastatin increased *Drosophila* lifespan

**Figure 2:**
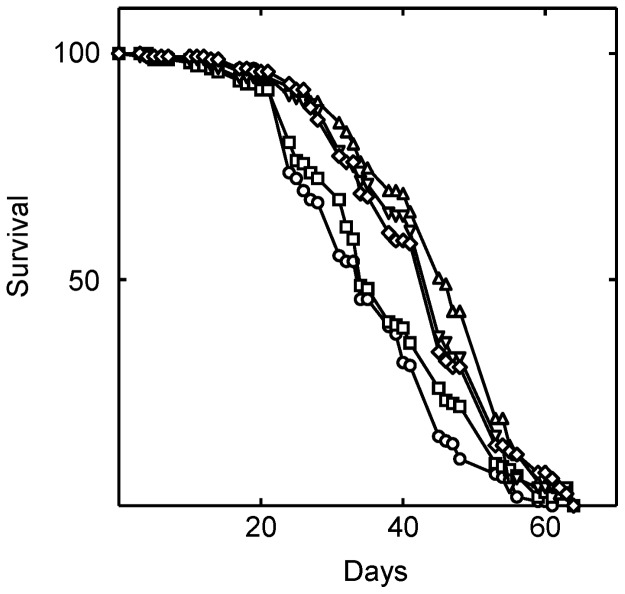
Simvastatin extends the lifespan of *Drosophila*. Simvastatin was fed to the flies in their food beginning on the first day of eclosure. Shown is survival at zero (○), 0.024 (□), 0.24 (▵), 2.4 (▿), and 12 (⋄) mM simvastatin. As judged by the log rank test, the lifespan of the flies was significantly changed when they were treated with 24 µM (P = 0.047), 240 µM (P<0.0001), 2.4 mM (P = 0.017) and 12 mM (P = 0.0004) simvastatin.

We found that simvastatin dose-responsively increased *Drosophila* lifespan by 25% (P<0.0001; Figure 2; Table 1). Two hundred forty µM simvastatin optimally increased lifespan, while higher or lower doses were less effective (Table 1). Thus, it appears that partial inhibition of HMG-CoA reductase activity increases *Drosophila* lifespan.

**Table 1:** Effect of drug treatments on the lifespan of *Drosophila* in Figures 2, 3, 4.

Lifespan data from Figure 2		
Treatment	Mean lifespan (days)	Standard error
Vehicle	35.1	0.8
Simvastatin (0.024 mM)	36.8	1.0
Simvastatin (0.24 mM)	43.9	1.0
Simvastatin (2.4 mM)	37.7	0.9
Simvastatin (12 mM)	38.3	0.9
Lifespan data from Figure 3		
Treatment	Mean lifespan (days)	Standard error
Vehicle	24.3	0.3
Simvastatin (240 µM )	25.5	0.3
Methoprene (320 µM )	25.7	0.4
Simvastatin (240 µM) and methoprene (320 µM)	28.0	0.6
Lifespan data from Figure 4		
Treatment	Mean lifespan (days)	Standard error
Vehicle	26.5	0.4
Simvastatin (240 µM)	29.7	0.4
CoQ10 (5 mM)	24.3	0.5
CoQ10 (5 mM) and simvastatin (240 µM)	28.4	0.5

### Statins do not extend lifespan by reducing caloric consumption (CR) or locomotor activity

Caloric restriction can extend the life- and healthspan of many metazoans [14]. Modified CAFE assays [16] and assays we termed “fecal plaque assays” (FPAs) [17], [18] were used to determine whether reduced food consumption might be the source of the longevity effects of simvastatin [19] (see *Materials and Methods*). CAFE assays measure the volume of liquid medium consumed from micropipette “straws”. FPAs measure the deposition of fecal waste in discrete “plaques” on the inner walls of flybottles. Both assays were utilized because CAFE assays are widely used, while FPAs are performed under conditions similar to those used for our lifespan assays. There is a strong correlation between food consumption data generated by the two assays [20]. Simvastatin either increased or had no effect on food consumption as measured by CAFE assays (Table 2). The drug also had no effect on food consumption as measured by FPAs (Tables 3 and 4). There was no effect on fecal plaque number or size. Simvastatin also had no effect on the weight of the flies (Table 5). Thus, the longevity effects of simvastatin do not involve reduced food intake.

**Table 2:** Food consumption does not change in response to chemical additions to the diet as determined by CAFE assays analyzed with Tukey's Multiple Comparison Testa.

Treatment	Mean Difference^b^	Q	Summary^c^	95% Confidence interval of difference
Control vs. simvastatin	−0.2868	6.652	**	−0.4678 to −0.1058
Control vs. methoprene	−0.1455	3.374	NS	−0.3265 to 0.0356
Control vs. Methoprene + simvastatin	−0.1475	3.421	NS	−0.3285 to 0.0336
Simvastatin vs. methoprene	0.1413	3.278	NS	−0.0397 to 0.3224
Simvastatin vs. methoprene + simvastatin	0.1393	3.231	NS	−0.0417 to 0.3204
Methoprene vs. methoprene + simvastatin	−0.0020	0.0467	NSMaterials and Methods	−0.1831 to 0.1790

a See *Materials and Methods* for specifics regarding the studies. The fly medium contained either an equal volume of vehicle, 2.4 mM simvastatin, 320 µM methoprene, or their combination.

b Mean difference between food consumption in the absence and presence of the drug(s).

c The notation ** indicates the difference was highly significant (P≤0.01). The notation NS indicates that the results were not significantly different.

**Table 3:** Food consumption does not change in response to chemical additions to the diet as determined by both FPA and CAFE assays.

Treatment^1^	FPAs		CAFE Assays
	Plaque number/cm^2^/fly (mean ± SD)	Plaque Diameter (mm)^2^ (mean ± SD)	μL consumed/fly/hr (mean ± SD)
Control	0.107±0.003^a2^	0.089±0.003^a^	0.343±0.010^a^
Simvastatin	0.108±0.008^a^	0.093±0.003^a^	0.472±0.026^b^
L-774832	0.112±0.009^a^	0.086±0.003^a^	0.386±0.010^a^
GGTI-298	0.107±0.005^a^	0.094±0.003^a^	0.474±0.049^b^

1 See *Materials and Methods* for specifics regarding the studies. The medium contained either an equal volume of vehicle, 2.4 mM simvastatin, 320 µM methoprene, 20 µM L744832, or 300 µM GGTI-298.

2 Column values with the same superscript letters are not significantly different, as determined by one way ANOVA followed by Tukey's multiple comparison test. Column values with different superscript letters are significantly different (P≤0.05). See *Materials and Methods* for specifics regarding the studies.

**Table 4:** Food consumption as measured by FPA does not change in response to simvastatin or methoprene.

Control^a^	Simvastatin	Methoprene	Simvastatin plus Methoprene
0.1008±0.0062^a^	0.0868±0.0135^a^	0.1288±0.0122^a^	0.1062±0.0047^a^

a These data are the means plus or minus the standard error of the plaque number per cm^2^ per fly per hour. The plaque values are not significantly different as judged by ANOVA followed by Tukey's multiple comparison test. See *Materials and Methods* for specifics regarding the studies.

**Table 5:** Fly weight in response to drug treatments.

Treatment^1^	Weight^2^ (mg/10 flies)
Vehicle	8.22±0.17^a^
Simvastatin	8.23±0.05^a^
Methoprene	8.07±0.11^a^
L-774832	8.12±0.06^a^
GGTI-298	8.08±0.09^a^

1 Newly eclosed Drosophila were maintained for 12 days at 25°C with medium containing either an equal volume of vehicle, 2.4 mM simvastatin, 320 µM methoprene, 20 µM L744832, or 300 µM GGTI-298. Groups of 10 flies were anesthetized with CO_2_, immediately removed from the flybottles, and weighed (n = 12).

2 Column values with the same superscript letters are not significantly different, as determined by one way ANOVA. See *Materials and Methods* for specifics regarding the studies.

A drug-induced change in locomotion might alter longevity [21]. However, we found no effect of simvastatin on locomotor activity using an assay which closely recapitulates the conditions of the lifespan assays (Table 6). Thus, simvastatin extended *Drosophila* longevity without the confounding effects of CR or altered locomotion.

**Table 6:** Locomotor activity of simvastatin treated *Drosophila*

Treatment1	N	Mean±SEM^2^	P value
Control	5	15,302±2683	
Simvastatin	5	15,105±3486	P = 0.6244

1 Groups of 10 newly eclosed Drosophila were maintained for 14 days at 25°C with medium containing either an equal volume of vehicle or 2.4 mM simvastatin in a TriKinetics Drosophila Activity Monitoring System.

2 Infrared beam disruptions per 24 hours.

### Statins do not extend lifespan reducing juvenile hormone (JH) signaling

**Figure 3:**
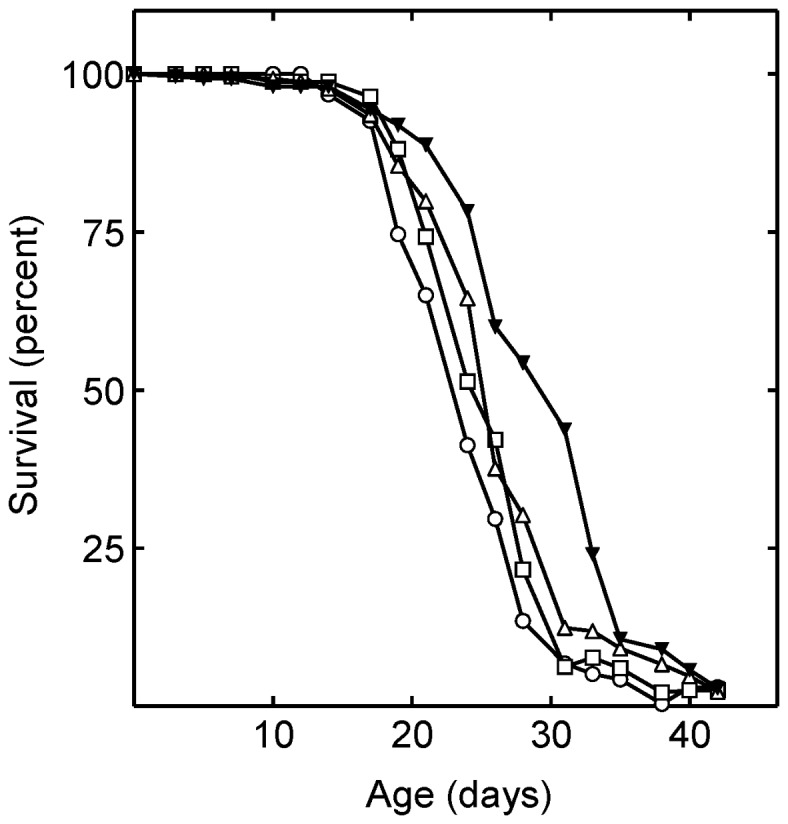
Juvenile hormone signaling and simvastatin treatment stimulate lifespan synergistically. Shown are the lifespans of *Drosophila* in the absence of drugs [control (○)]; and in the presence of 240 µM simvastatin (□); 320 µM methoprene (▵); and 320 µM methoprene and 240 µM simvastatin together (▾). The mean lifespan of the simvastatin, methoprene, and simvastatin with methoprene treated flies was significantly increased (P = 0.02, P = 0.0034, and P<0.0001, respectively).

The synthesis of mevalonate from acetyl CoA is irreversible (Figure 1). Thus, in insects, mevalonate is committed to the synthesis of non-cholesterol end-products of the isoprenoid pathway. We were unable to reliably measure JH levels using liquid chromatography tandem mass spectrometry (LC-MS/MS). For this reason, we investigated the role of JH signaling in longevity by treating flies with methoprene, a highly specific, stable, small molecule JH receptor agonist widely used to prevent insect molting (Figure 3). Methoprene and simvastatin each increased lifespan when administered alone, and together their effects were synergistic (Figure 3; Table 1). These results indicate that simvastatin does not increase *Drosophila* lifespan by reducing JH receptor signaling. Further, because their effects are synergistic at their optimum dosages, simvastatin and methoprene likely utilize independent pathways to increase lifespan.

Neither methoprene alone, nor methoprene and simvastatin together had any significant effect on food consumption as determined using CAFE assays or FPAs (Tables 2, 3 and 4). In addition, methoprene had no effect on the weight of the flies (Table 5). Thus, the longevity effects of methoprene are not due to induced CR.

### Statins do not extend lifespan by reducing endogenous CoQ10

**Figure 4:**
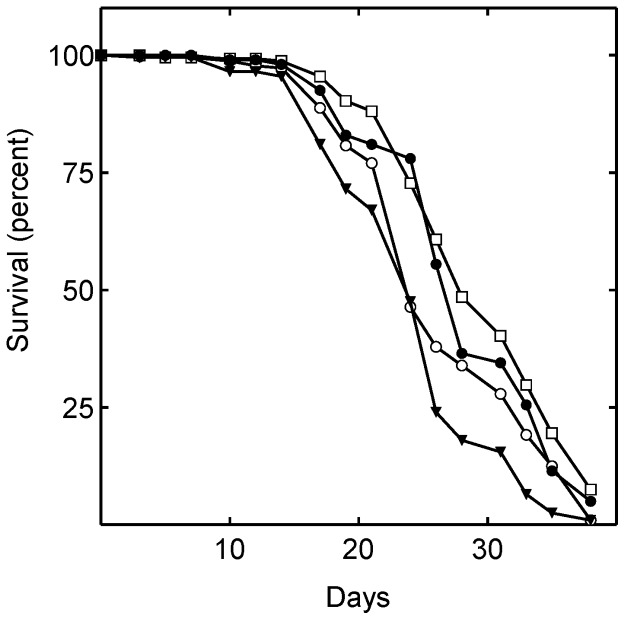
Ubiquinone administration slightly reduces lifespan. Shown are the lifespan of flies consuming food containing no drugs [control (○)]; 240 µM simvastatin (□); 5 mM CoQ10 (▾); 5 mM CoQ10 and 240 µM simvastatin (•). The mean lifespans of the flies treated with simvastatin alone (P<0.0001) and with 5 mM CoQ10 plus simvastatin (P = 0.0058) were significantly greater than that of control. The mean lifespan of the flies treated with 5 mM CoQ10 alone was significantly less than control (P = 0.0001)

Because mevalonate is a precursor in ubiquinone biosynthesis (Figure 1), we investigated whether simvastatin extended lifespan by reducing endogenous ubiquinone levels. Diets supplemented with simvastatin and/or ubiquinone were administered to *Drosophila*. Supplementation with CoQ10 alone significantly shortened lifespan, while simvastatin alone significantly lengthened it (Figure 4; Table 1). CoQ10 and simvastatin together significantly lengthened lifespan, although not by as much as simvastatin alone (Figure 4; Table 1).

**Figure 5:**
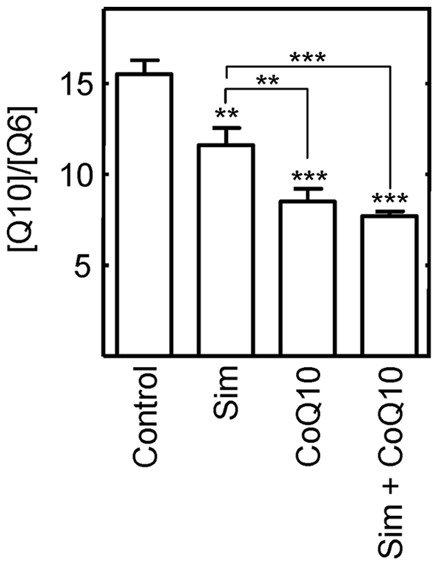
Supplementation with simvastatin and CoQ10 reduces endogenous ubiquinone levels. Ubiquinone (5 mM) and simvastatin (240 µM) administered to *Drosophila* in their food alone or together decreased the endogenous levels of CoQ10 as measured by LC-MS/MS. Two asterisks indicate the differences were highly significant (P≤0.01) and three indicate that the differences were very highly significant (P≤0.001). *Sim* below a bar indicates the flies were treated with simvastatin.

To investigate whether effects on endogenous CoQ10 levels might explain the results in Figure 4, we quantified the effects of the drugs on the endogenous levels of CoQ in *Drosophila* by LC-MS/MS. Simvastatin treatment significantly decreased internal CoQ10 levels by approximately 21% (Figure 5), consistent with inhibition of mevalonate production (Figure 1). Surprisingly, supplementation with CoQ10 also significantly decreased internal CoQ10 levels, in this case to 53% of control levels (Figure 5). Simvastatin and CoQ10 together decreased internal CoQ levels to 47% of the level in untreated flies.

Therefore, the lifespan effects of simvastatin and CoQ10 do not correlate with their effects on endogenous CoQ10 levels. These results strongly indicate that simvastatin does not extend *Drosophila* lifespan by decreasing the level of endogenous CoQ10.

### Statins decreased the active, farnesylated and geranylgeranylated forms of specific RAS-related signaling GTPases

The isoprenoids synthesized from mevalonate are used for the prenylation of the small Ras-related signaling GTPases. These modifications are essential for their activity (see Discussion). Statin-induced changes in the prenylation of these small signaling proteins are thought to be responsible for the non-cholesterol-related effects of statins in humans [8], although the vast majority of the studies supporting this idea have been performed using cultured cells [8]. Thus, it is still unclear whether changes in protein prenylation are responsible for the non-cholesterol related effects of statins on human lifespan.

**Figure 6:**
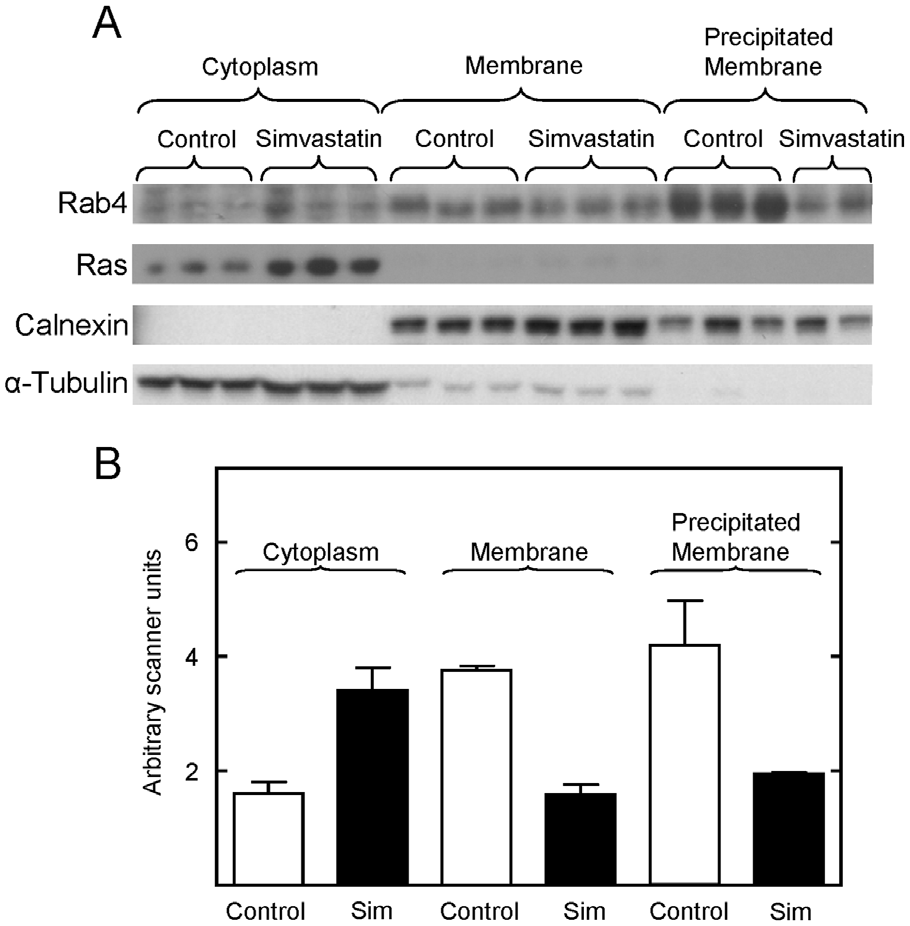
Simvastatin administration to mice decreased prenylation of specific small G proteins, as measured by a decrease in their membrane association. Panel A, Western blot showing the level of Rab4, Ras, calnexin, and α-tubulin in the cytoplasmic and membrane fractions purified from the liver of control and simvastatin treated mice. Panel B shows the quantification of the data shown in Panel A. The levels of Rab4 in the cytoplasmic fractions were normalized to the level of α-tubulin in each sample. The levels of Rab 4 in the membrane fractions were normalized to the level of calnexin in each sample. Means and standard errors obtained with tissue from control (open bars) and simvastatin treated (closed bars) mice are shown. One of the precipitated membrane samples was overloaded on this blot, and the amount of Rab4 reported in panel B was determined from another blot. The scanner units were adjusted to facilitate comparisons between membrane and cytoplasmic fractions.

To investigate this question, we first examined whether statin treatment of intact animals affected the prenylation, and therefore the membrane localization of small Ras family GTPases. After multiple attempts, the antibodies available did not reproducibly detect these proteins on Western blots of membrane and cytoplasmic proteins from *Drosophila*. However, we were able to detect many of these proteins in liver membrane and cytoplasmic fractions prepared from statin treated mice. Simvastatin treatment depleted the active membrane bound form of Rab4 and increased the level of its inactive cytoplasmic form (Figure 6A and B). Similarly, simvastatin treatment increased the cytoplasmic level of inactive Ras by 3.3-fold. The membrane bound form of Ras was below the level of detection in these studies. In contrast, simvastatin had no effect on the membrane bound and cytoplasmic forms of Rac1/2/3 (data not shown). Importantly, calnexin, a membrane-specific protein, and α-tubulin, a cytoplasmic protein, partitioned appropriately during cell fractionation, confirming that the procedures effectively separated their cellular compartments (Figure 6A). Together, these results show that simvastatin reduced the prenylation of specific Ras GTPases in vivo, leading to the accumulation of their inactive forms in the cytoplasm.

### Inhibition of isoprenyl transferases extended *Drosophila* lifespan

**Figure 7:**
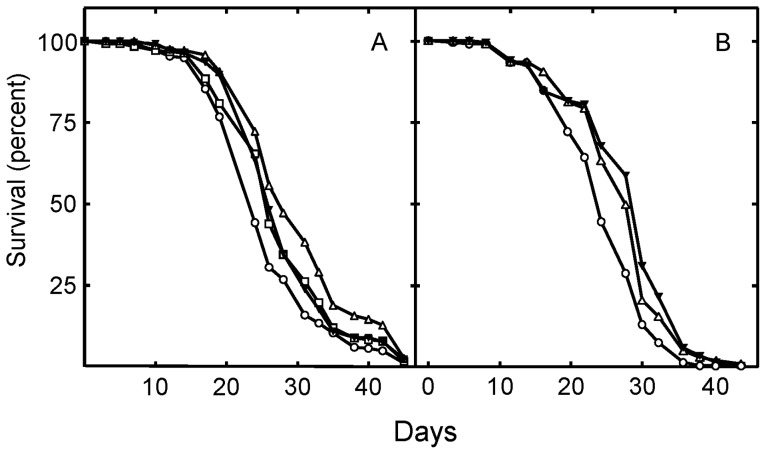
Inhibition of farnesyl transferase or geranylgeranyl transferase I activity increases *Drosophila* lifespan. Shown in panel A are the lifespans of *Drosophila* in the absence of drugs [control (○)]; and in the presence of 5 µM (□); 10 µM (▵); or 20 µM (▾) L744832. The mean lifespan of flies treated with 5, 10, and 20 µM L744832 were significantly increased relative to control (P = 0.0089, P<0.0001, and P = 0.0019, respectively). In panel B are shown the lifespans of *Drosophila* in the absence of drugs [control (○)]; and in the presence of 100 µM (▵) or 300 µM (▾) GGTI-298. The mean lifespan of flies treated with 100 and 300 µM GGTI-298 was significantly increased relative to control (P<0.0001 and P<0.0001, respectively).

To investigate whether drug induced reduction in protein prenylation could be responsible for the increased longevity found with statins, the effects of specific inhibitors of protein prenylation on *Drosophila* lifespan were investigated. Both L744832, a potent and specific farnesyl transferase inhibitor (Figure 7A), and GGTI-298, a potent and specific type I geranylgenranyl transferase inhibitor, significantly increased *Drosophila* lifespan in a dose-responsive manner (Figure 7B). Further, neither drug had any significant effect on food intake as measured by fecal plaque number and size (Table 3), or fly weight (Table 5). CAFE assays detected an increase in food consumption with both simvastatin and GGTI-298 treatments, while L744832 produced no effect (Table 3). Together these results indicate that the increase in lifespan induced by the transferase inhibitors are not due to induced CR. Likewise, neither L744832, GGTI-298, nor simvastatin had any significant effect on the movement of the flies (Tables 6 and 7). Two independent studies with GGTI-298, and 4 independent studies with L744832 found no effect on movement. These results precluding altered locomotor activity as a source of the longevity effects of the drugs.

**Table 7:** Locomotor activity of L744832 and GGTI-298 treated *Drosophila*.

Treatment1	N	Mean ± SEM	P value	
**Study 1**				
Control	4	12,939±692.4		
GGTI-298	4	13,547±4272		0.8952
**Study 2**				
Control	9	20,384±2271		
L744832	9	16,961±1969		0.2715

1 Groups of 10 newly eclosed Drosophila were maintained for 14 days at 25°C with medium containing either an equal volume of vehicle, 20 µM L744832, or 300 µM GGTI-298 in a Drosophila Activity Monitoring System.

The effects of L744832 and GGTI-298 on lifespan were not additive, and co-treatment with simvastatin did not lead to greater lifespan extension than treatment with simvastatin alone (data not shown). These results suggest that simvastatin extends lifespan by inhibiting farnesylation and geranylgeranylation. The results are consistent with the apparent increase in human lifespan associated with statin treatment of healthy, normocholesterolemic adults [3].

### Simvastatin decreased arrhythmias in old flies

**Figure 8:**
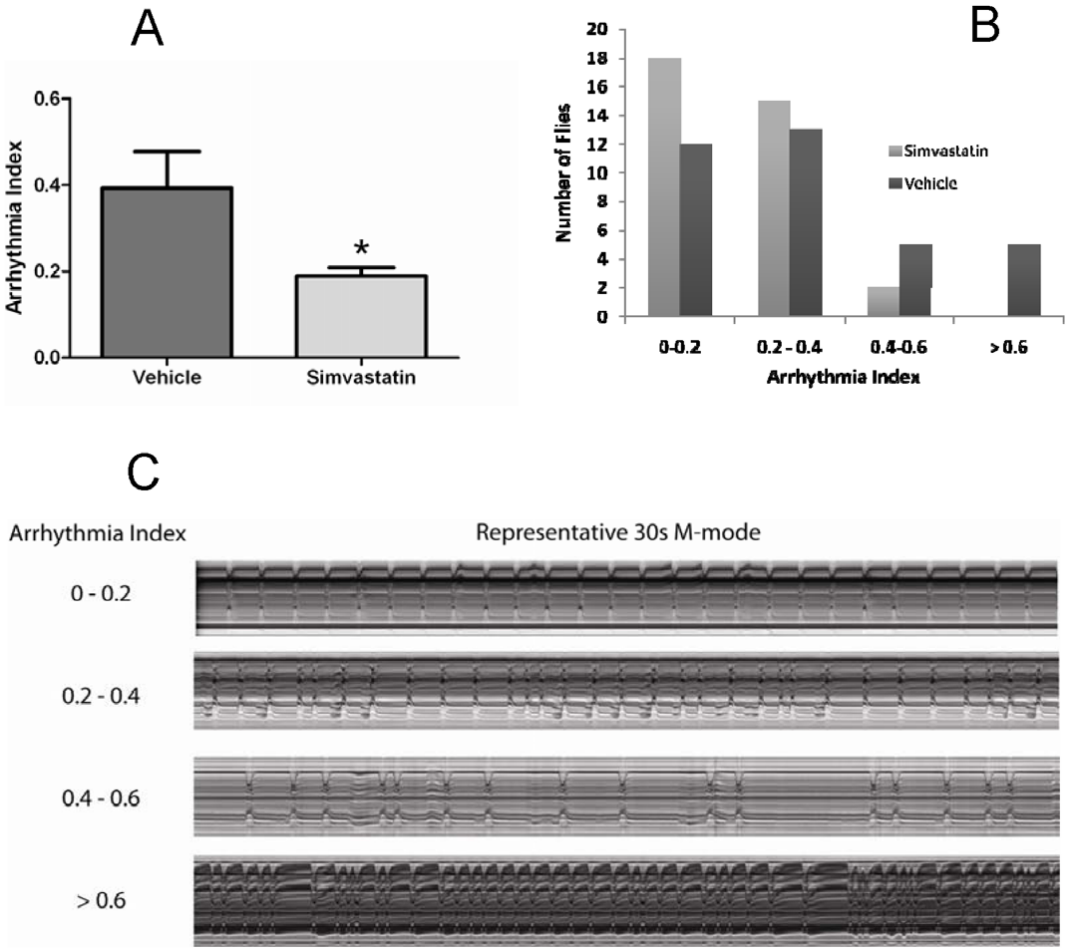
Simvastatin administration reduces the number of old flies that exhibit severe arrhythmias, as quantified by the AI, a measure of heartbeat regularity. Panel A shows a summary of the results showing that AI is significantly reduced in hearts from 4 week old flies fed simvastatin compared to vehicle (n = 35 flies for each group; p<0.05, as judged by an unpaired t-test). Panel B, the number of simvastatin and vehicle treated flies exhibiting each arrhythmia index is shown. Panel C, examples of M-mode intervals representing different arrhythmicity indices.

To investigate the mechanisms for the positive effects of simvastatin on lifespan, its effects on cardiac function were studied using 4-week-old flies. Analysis of high speed movies of beating hearts showed that simvastatin dramatically reduced the overall incidence of arrhythmias in these old flies (Figure 8). The arrhythmia index (AI) of 0.39 in untreated 4-week old flies is similar to that previously reported (Ocorr et al., 2007). However, simvastatin treatment with the same concentration that extended lifespan (Figure 2) lowered the AI to half (0.19), which is typical of young hearts. Simvastatin treatment had no effect on systolic interval length (0.30±0.02 sec for control v. 0.31±0.01 sec for simvastatin, mean ± SEM, P = 0.99 ) nor did it affect the % fractional shortening (42±1% control vs. 42±1% simvastatin, mean ± SEM, P = 0.8 ), which is a measure of heart contractility. Interestingly, the diastolic interval (DI) length was reduced in the simvastatin group (1.01±0.09 sec for control vs. 0.79±0.07 sec for simvastatin, mean ± SEM ) but this decrease did not reach significance (P = 0.065). Nevertheless, because the systolic interval length remained unchanged, this decrease in DI resulted in a significant increase in the amount of time the heart is contracted during each contraction/relaxation cycle: 0.26±0.02 sec/ heart period for control vs. 0.3±0.02 sec/ heart period for simvastatin treated flies (mean ± SEM, P = 0.035). Thus, the longevity effects of simvastatin in flies may result from a reduction in arrhythmias and an increase in the relative time the heart spends in contraction.

### Endogenous simvastatin levels

We hoped to compare the endogenous levels of simvastatin in flies with that of treated animals. However, repeated attempts to measure the level of simvastatin in isolated hemolymph and serum from simvastatin treated mice were unsuccessful using two established extraction techniques [22], [23], and GC-MS/MS (*Materials and Methods*). These results were probably due to the low levels of the drug in the serum and hemolymph, and the limited quantities of each available. For example, each fly yielded approximately 0.5 μL of hemolymph.

### Statin treatment did not alter stress resistance

In flies and mammals, interventions that extend lifespan often enhance resistance to various stresses [24]–[26]. However, we were unable to detect any effect of simvastatin on resistance to anoxia-reoxygenation stress or paraquat toxicity using several standard techniques with either young or old flies (*Materials and Methods*). Thus, despite enhancement of cardiac performance and lifespan, statins do not appear to increase general stress resistance.

## Discussion

A number of novel results are presented here. First, we show that simvastatin extended the lifespan of *Drosophila,* and increased cardiac functioning in old flies. Second, we demonstrated that statin treatment reduced protein prenylation of specific Ras signaling GTPases in mice. Third, we showed that drugs which reduce protein prenylation in vivo extend the lifespan of *Drosophila*. As a part of these studies, we also showed that juvenile hormone agonists extend, rather than decrease the lifespan of adult *Drosophila*, and that CoQ10 supplementation reduces, rather than increases, internal CoQ10 levels in *Drosophila*.

### Lifespan extension by statin treatment does not involve reduced ubiquinone biosynthesis

While simvastatin treatment reduced internal ubiquinone levels, our data appear to exclude this effect as the mechanism for increased fly longevity. Dietary CoQ10 actually decreased internal CoQ10 levels. We can only speculate at this time about the reason for this effect. *Drosophila* may have a mechanism for sensing dietary ubiquinone levels, and downregulating internal levels in response.

Statin treatment is associated with reductions in blood and tissue ubiquinone levels in statin treated humans, rats, and mice [27]–[29]. As mentioned in the introduction, statin treatment also reduces all-cause mortality in healthy people with no history of cardiovascular disease [3]. Here we found that these two phenomena are unrelated in *Drosophila.* Endogenous reduction in ubiquinone induced by CoQ10 feeding shortened lifespan, and may have blunted the beneficial effects of simvastatin on lifespan (Figs. 5 and 6). Thus, reduced ubiquinone levels do not appear to contribute to the salutary effects of statins, and may oppose them. Indeed, feeding *Drosophila* yeast lacking CoQ reportedly reduces their lifespan relative to flies fed CoQ replete yeast [30]. In rodents and C. elegans, most [31]–[34], although not all [35], [36], studies find that CoQ10 supplementation has no effect on lifespan. Taken together, the results presented here and published data indicate that reduced ubiquinone levels are not responsible for the longevity effects of simvastatin.

### JH and lifespan in *Drosophila*

The administration of methoprene on a wick of paper rescued the longevity phenotype (reduced the lifespan) of daf-2 mutant *Drosophila* [37], [38]. Tatar and his colleagues concluded from this that JH deficiency extends *Drosophila* longevity. Our studies with wild type Oregon-R flies found that methoprene in food reproducibly increased lifespan (Figure 3). The reasons for the apparent differences in these results are unclear. However, the data in the Tatar study involved a mutant fly. Our results make it very unlikely that simvastatin increases the lifespan of wild type *Drosophila* by decreasing JH signaling.

### Inhibition of protein isoprenylation increases longevity

Ras and Rho farnesylation, and Ras, Rho, and Rab geranylgeranylation anchor these proteins on the inner side of the cytoplasmic membrane, where they can interact with downstream effectors of cytoplasmic signaling networks [6]. Statins induce cholesterol-independent effects which include enhanced endothelial function, greater stability of atherosclerotic plaque, decreased oxidative stress and inflammation, and reduced thrombogenesis [39]. Many of these effects appear to be mediated by inhibition of isoprenoid biosynthesis [8]. The Ras family of small signaling GTPases are isoprenylated at their C-termini, which allows them to cycle between a membrane tethered, active state, and a soluble, inactive state [8], [40]. Statin inhibition of isoprenoid biosynthesis reduces the number of membrane-bound, active proteins, and increases the pool of soluble, inactive proteins [8].

Most studies of the effects of statins on protein isoprenylation have been performed in vitro [8], [41]. Our in vivo studies found that Rab4 and Ras respond to simvastatin treatment by increasing the level of their inactive, soluble forms (Figure 6). The Rab GTPases are a family of 63 mammalian proteins which control secretion, endocytosis, signal transduction and development [42]. Rab4 regulates specific steps in vesicle trafficking and receptor recycling in early endosomes. Thus, reduced Rab4 activity could influence the secretion and/or display of other signaling factors and membrane receptors. The Ras GTPases are a family of 4 proteins which link tyrosine kinase activation at the cell membrane to growth, differentiation, apoptosis, cytoskeletal organization and membrane trafficking [43]. Thus, simvastatin may pleiotropically alter signal transduction by multiple systems. We used geranylgeranyl and farnesyl transferase inhibitors to show that such effects on signal transduction can increase lifespan. This is the first demonstration that these pathways can increase lifespan.

### Statin treatment improved cardiac functioning

We showed that simvastatin enhances cardiac functioning in *Drosophila* by reducing the age-related increase in arrhythmias (Figure 8). Simvastatin also increased pumping efficacy by decreasing the DI and increasing the relative proportion of the cardiac cycle spent in contraction (Figure 8). These results are consistent with reports suggesting that statins are antiarrhythmic and inhibit atrial fibrillation in patients with coronary artery disease [44], [45]. In humans and animal models, statins protect the myocardium from acute ischemia – reperfusion injury and increase survival after stroke (reviewed in [46]) These effects are mediated by inhibition of HMG-CoA reductase activity, but appear to involve isoprenoid, rather than cholesterol biosynthesis (reviewed in [47], [48]).

### Conclusions

We directly showed that statin treatment increased the lifespan and cardiac health of *Drosophila*. We also showed that statin treatment decreased protein isoprenylation. Further, we showed that decreased protein isoprenylation extended the lifespan of *Drosophila*. The linkage between these phenomena indicates that statins can extend lifespan by decreasing protein prenylation, which leads to enhanced cardiac health in old age and increased longevity.

## Materials and Methods

### *Drosophila* survival studies

Male flies were used for these studies to avoid the confounds associated with redirecting metabolic energy from survival to reproductive behavior. Freshly eclosed flies (Wild-type Oregon-R-C *Drosophila Melanogaster* (Bloomington *Drosophila* Stock Center, Department of Biology, Indiana University, Bloomington, IN) were sorted under light CO_2_ anesthesia, and male flies placed into 8 ounce fly bottles (Genesee Scientific) and covered with 35×10 mm Petri dish lids (Falcon) containing ∼5.5 ml of Standard Cornmeal Medium (SCM). The concentrations of drugs indicated in the figure legends were dissolved in DMSO and 10 µl applied to the surface of the SCM and spread carefully over the entire surface. Controls utilized 10 µl of DMSO, spread as described above. L744832 was from Enzo (catalogue #G-242) and GGTI-298 from Sigma (catalogue #G5169). Dye studies indicate that 10 μL of DMSO diffuses to approximately 1 mm depth over 48 hours. Unless otherwise indicated, a total of 4 bottles per drug concentration, with 50 male flies per bottle (200 flies total) were used for each determination. The lids were attached to the bottle opening and secured with tape. The walls of the plastic bottles were pierced with small air holes. The bottles were incubated with the lid closures on top (to minimize evaporative drying) at 25°C, 60% humidity, and with a 12 ∶12 hour light: dark cycle. The SCM-lids were replaced twice weekly. The number of flies living at each time point was determined by visual inspection. CO_2_ anesthesia was not used after the initial sorting of males from females.

### Quantification of food consumption

The capillary feeder (CAFE) assay [16] was modified to use 30 flies in eight ounce plastic fly bottles with four, 100 µl graduated glass microcapillary pipettes containing 5% sucrose, 5% autolyzed yeast extract (Fisher Scientific, Pittsburgh, PA), and 0.01% (v/v) DMSO either alone (control) or containing the concentration of drug indicated in the text. A small amount of red food coloring was added to facilitate measurements. The cotton bottle closures were saturated with 25 ml of reverse osmosis, nano-pure water (Barnstead, Inc.), to maintain humidity during the 24 hour incubation at 25°C. Food intake was also measured using FPAs. The standard SCM with agar in Petri dish lids were prepared as described. One-half ml of SCM without agar was mixed with 10 µl of DMSO without (control) or with 10 µl of the indicated concentration of drug and one drop of red food color, and evenly spread over the surface of an SCM lid. The lids were used to feed bottles of 50 flies for 24 hours. After removal of the lids and the flies, 5, 4×4 cm square regions were randomly marked on the side of each bottle, and the numbers of red feces dots (plaques) counted. Four bottles were used per control or treatment group. To determine plaque sizes, fly bottles from FPAs were positioned under a Celestron Handheld Digital Microscope, Model #44302-A, and their diameter determined using the software provided (n = 40 for each condition). The mean number of plaques per square centimeter and plaque diameter was compared by t-test or one way ANOVA using GraphPad Prism.

### Quantitation of locomotor activity

*Drosophila* movement was quantified using a Trikinetics Drosophila Activity Monitoring System and DAMSystem data acquisition software package sold with the unit. The system records the number of disruptions of three infrared beams by moving flies. Drug treatment was begun immediately after eclosure. After 14 days of treatment, the number of disruptions caused by 10 flies was recorded continuously for three days. The total number of disruptions of the beams per 24 hours is reported.

### *Drosophila* cardiac function

Male flies were collected at eclosion and aged for 4 weeks at 25^o^C on SCM in the presence or absence of 240 µM simvastatin, as described above. Heart physiology in flies was assessed using a semi-intact adult fly preparation combined with a semiautomated optical heartbeat analysis program that quantifies heartbeat parameters including diastolic and systolic diameters, heart period and rate, heart beat regularity and fractional shortening [49], [50]. In addition, the arrhythmia index, a measure of heartbeat regularity, is calculated from high-speed digital movies using the mean standard deviation of the heart period normalized to the median heart period. M-modes, a qualitative record showing heart edge movement over time, were also produced using the optical heartbeat analysis program [50].

### Quantitation of prenylated and soluble Rac1/2/3, Rab4 and Ras

Male C3B6F1 mice were maintained and drug treated as described [19]. Liver tissue from 18 month-old control and simvastatin treated mice (188 mg/kg diet) was snap frozen and stored in liquid nitrogen. Forty mg of tissue was powdered under liquid nitrogen with a mortar and pestle. Soluble and membrane bound proteins were prepared using a ProteoJET Membrane Protein Extraction Kit (www.fermentas.com) as described by the supplier, except that the Cell Permeabilization and Membrane Protein Extraction Buffers contained protease inhibitor cocktail (10 µl per ml; Sigma). Western blots were performed using standard procedures, and immobilized proteins were visualized using IgG against α-tubulin, Rac1/2/3, Rab4, and Ras from Cell Signaling, and antibody against calnexin and goat anti-rabbit IgG conjugated to horseradish peroxidase from Abcam.

### LC-MS/MS analysis of endogenous CoQ10

Samples were prepared as described [23]. Briefly, 300 to 400 flies were homogenized with 1 ml of water and 50 μL of 10 mg/ml ethanolic butylated hydroxytoluene using an Ultra-Turrax homogenizer (Tekmar Company). The homogenate was mixed with 1 ml of 0.1M aqueous SDS, and 2 ml of reagent alcohol and vortexed for 30 s. The mixture was combined with 2 ml of hexane and vigorously vortexed for 2 min followed by 5 min centrifugation at 1000×g. One ml of the hexane layer was transferred to a small vial and dried under argon. A working solution of 800 μg/mL CoQ10 (Cat # C9538, Sigma-Aldrich, St. Louis, MO) in hexane and an internal standard solution of 600 μg/mL CoQ6 (Cat # 900150-0 from Avanti Polar Lipid, Inc., Alabaster, AL) in ethanol were prepared. CoQ10 calibration solutions were prepared at 0, 0.025, 0.05, 0.1, 0.5, 1, 2, 20, 150, and 500 μg/mL by serial dilution of the CoQ10 working solution with 50% ethanol. Each dilution was spiked with CoQ6 internal standard to a final concentration of 6 μg/mL. LC-MS/MS analysis was performed on an Agilent 6510 Q-TOF system coupled with an Agilent HPLC-Chip Cube MS interface (Agilent Technologies, Santa Clara, CA). Sample injection, enrichment, desalting, and HPLC separation were carried out on the Agilent HPLC Chip with an integrated trapping column (160 nL) and a separation column (Zorbax 300SB-C18, 75 μm ×150 mm, 5 μm in particle size). The samples were first loaded onto a trapping column with a solvent mixture of 2-propanol (HPLC grade)/methanol (HPLC grade)/formic acid (Fisher Scientific) (55:45:0.05, v/v) with 5 mmol/L methylamine at a flow rate of 2.8 μL/min (Agilent 1200 series capillary pump). The analytes were separated isocratically using the same mobile phase at a flow rate of 300 nL/min. The Chip spray voltage was set as 1950 V and varied depending on the chip conditions. The temperature and flow rate of the drying gas were 325°C and 4 L/min. Nitrogen was the collision gas. The collision energy followed the equation with a slope of 3 V/100 Da and an offset of 2.5 V. MS/MS experiments were carried out by monitoring the fragmentations of the methylammonium adduct ([M+CH_3_NH_3_]^+^) of the analyte and internal standard molecules. A calibration curve for CoQ10 quantification was constructed by plotting the ratios of areas of peaks found in the selected-ion chromatograms for monitoring the transitions of CoQ10 (*m/z* 894.740 197.075) over CoQ6 (*m/z* 622.483 197.075) versus the molar ratios of CoQ10 over CoQ6. The amount of CoQ10 in the samples were subsequently determined from the peak area ratios found in the selected-ion chromatograms for the same transitions of CoQ10 over CoQ6, the calibration curve, and the amount of CoQ6 spiked into the samples.

### LC-MS/MS quantification of simvastatin in serum and hemolymph

Two methods were utilized for sample preparation for LC-MS/MS analysis. In the first, 200 μL of serum from a simvastatin-treated mouse, or hemolymph from 70 simvastatin-treated flies extracted into a final volume of 200 μL with PBS, were spiked with an internal standard of 1.8 nmol of lovastatin, and extracted as described [22]. Briefly, the samples were mixed with 120 μl of ammonium acetate (100mM, pH4.5), vortexed, loaded onto 0.3 ml Chem Elut extraction cartridge (Varian Sample Preparation Products, Harbor City, CA, USA), and incubated and eluted according to the manufacturer's instructions. The eluents were dried under a stream of argon. In the second method, sample extraction was performed as described in [23]. Serum and hemolymph samples, as described above, were mixed with 1 ml of water, 50 µl of ethanolic butylated hydroxytoluene (10 mg/ml), 1 ml of 0.1M SDS, and briefly mixed by vortexing. Following the addition of 2 ml of reagent alcohol (Sigma), the solution was vortexed for 30 s. Following the addition of 2 ml of hexane, vortexing for 2 min, and centrifugation for 5 min at 1000 g, the hexane layer was transferred to a 15 ml tube and dried under a stream of argon gas. LC-MS/MS analysis of simvastatin and lovastatin was performed on a LTQ linear ion-trap mass spectrometer (Thermo Electron, San Jose, CA) coupled with an Agilent 1200 Series capillary HPLC pump (Agilent Technologies, Santa Clara, CA) and a Zorbax-SB-C18 column (0.5×150 mm, 5 μm in particle size, Agilent Technologies). Isocratic conditions of 65% B were used at a flow rate of 10 μL/min, where the mobile phases were A: 0.1% formic acid in water, and B: 0.1% formic acid in acetonitrile. The mass spectrometer was set up to monitor the fragmentation of the [M+H]+ ions of simvastatin (m/z 419) and lovastatin (m/z 405). The peak areas found in the selected ion chromatograms for the m/z 419 → 303 (for simvastatin) and m/z 405 → 303 ( for lovastatin) transitions were used to quantify simvastatin. The calibration curve was constructed by analyzing a series of standard solutions comprising of a 5.4 pmol/μl of lovastatin and 0, 50, 150, 250, and 500 pmol/μl of simvastatin under the same LC-MS/MS conditions.

### Anoxia/reoxygenation injury resistance

After 2 weeks of feeding either control or simvastatin supplemented food, a stream of argon was introduced through an opening in the bottom of 8 ounce plastic fly bottles containing 40 to 50 flies. Excess gas exhausted through the cotton closures at the top of the bottles. Flies almost immediately became motionless, and remained so during the 3.5 h of treatment. After the gas was discontinued, the cotton closure was temporarily removed and air was reintroduced into the bottle. Survival rates were determined 24 hours later. There were no deaths in the 2 hours immediately following reoxygenation.

### Dietary paraquat resistance

Two methods of determining the sensitivity of flies to dietary paraquat (a.k.a. Methyl viologen; N,N′-dimethyl-4,4′-bipyridinium dichloride). The first used fly bottles containing 4-day-old male flies incubated as described for lifespan studies with SCM containing lids spread with 10 µl of DMSO containing 1 or 5 mM paraquat, with or without 240 µM simvastatin. Control groups were cultured with SCM disks spread with 10 µl of DMSO or 10 µl of DMSO containing 240 µM simvastatin. The disks were changed twice weekly. A total of 4 to 8 bottles per treatment group, with 30–50 male flies per bottle (200 to 240 flies total) were used for each determination. Survival was assessed every other day. The second method was modified from published reports [16], [51]. Thirty-five to 45 flies in 8 ounce plastic fly bottles were fed standard SCM with or without 240 µM of simvastatin for 10 or 20 days. After 2–5 hours without food, Whatman filter paper wetted with 0.5 ml of 20 mM of paraquat in 5% sucrose was inserted into each bottle for 7 or 16 h, followed by 48 h under standard conditions. The number of survivors was determined by counting. Statistical significance was judged using two-tailed t-tests, as implemented in GraphPad Prism 5.0.

### Statistics

*Drosophila* survival analysis utilized the log-rank tests as implemented in SAS. The significance of the effects of simvastatin on survival after hypoxia-reoxygenation and paraquat stress, and on the effects of simvastatin on *Drosophila* heartbeat were determined using two tailed unpaired t-tests.
